# Effect of replacing polyol by organosolv and kraft lignin on the property and structure of rigid polyurethane foam

**DOI:** 10.1186/1754-6834-6-12

**Published:** 2013-01-28

**Authors:** Xuejun Pan, Jack N Saddler

**Affiliations:** 1 Department of Biological Systems Engineering, University of Wisconsin – Madison, 460 Henry Mall, Madison, WI 53706, USA; 2 Department of Wood Science, University of British Columbia, 2424 Main Mall, Vancouver, BC V6T 1Z4, Canada

**Keywords:** Kraft lignin, Lignin utilization, Organosolv lignin, Polyurethane, Rigid foam

## Abstract

**Background:**

Lignin is one of the three major components in plant cell walls, and it can be isolated (dissolved) from the cell wall in pretreatment or chemical pulping. However, there is a lack of high-value applications for lignin, and the commonest proposal for lignin is power and steam generation through combustion. Organosolv ethanol process is one of the effective pretreatment methods for woody biomass for cellulosic ethanol production, and kraft process is a dominant chemical pulping method in paper industry. In the present research, the lignins from organosolv pretreatment and kraft pulping were evaluated to replace polyol for producing rigid polyurethane foams (RPFs).

**Results:**

Petroleum-based polyol was replaced with hardwood ethanol organosolv lignin (HEL) or hardwood kraft lignin (HKL) from 25% to 70% (molar percentage) in preparing rigid polyurethane foam. The prepared foams contained 12-36% (w/w) HEL or 9-28% (w/w) HKL. The density, compressive strength, and cellular structure of the prepared foams were investigated and compared. Chain extenders were used to improve the properties of the RPFs.

**Conclusions:**

It was found that lignin was chemically crosslinked not just physically trapped in the rigid polyurethane foams. The lignin-containing foams had comparable structure and strength up to 25-30% (w/w) HEL or 19-23% (w/w) HKL addition. The results indicated that HEL performed much better in RPFs and could replace more polyol at the same strength than HKL because the former had a better miscibility with the polyol than the latter. Chain extender such as butanediol could improve the strength of lignin-containing RPFs.

## Background

Polyurethane is one of the most important synthetic polymers, and it is synthesized through a polyaddition reaction between a polyisocyanate (a polymeric molecule with two or more isocyanate groups, such as toluene diisocyanate (TDI) and methylene diphenyl diisocyanate (MDI)) and a polyol (a polymer with two or more reactive hydroxyl groups, such as polyethylene adipate and poly(tetramethylene ether)glycol). Both the polyisocyanates and the polyols are currently derived from petroleum oil. Polyurethane has varied applications in different areas from liquid coatings and paints, tough elastomers, rigid foams for packing and insulation, to flexible foam in mattress and car seats [1].

Lignin is one of the three major components in plant cell walls and the most abundant aromatic polymer in the nature [2]. Structurally, lignin is a 3-D networked polymer biosynthesized in plants from three monolignols, *p*-coumaryl alcohol, coniferyl alcohol, and sinapyl alcohol, through radical coupling processes [3]. Lignin plays a vital function in the plant’s defense system against degrading enzymes and diseases. The lignin also binds fibers together to form a strong and tough matrix of plants and provides mechanical support to the plant vessels for the transportation of water and nutrients [4]. However, the physical and chemical nature and functions of lignin make it troublesome in the utilization and conversion of lignocellulosic biomass. For example, lignin has to be removed (dissolved) during chemical pulping of wood to release/produce intact, strong, and bleachable fibers (pulp) for making paper. In bioconversion of lignocellulosic biomass to fuel ethanol, lignin is one of the major recalcitrance sources of the cellulosic substrates to cellulases. Furthermore, the lignin isolated from either chemical pulping or biorefining has not been utilized in a value-added way, and the most common lignin utilization is still steam and power production through combustion.

Extensive efforts have been made to explore high-value applications of lignin, in particular in polymeric materials, such phenolic and epoxy resins [5]. Considering the fact that lignin is a polymer with a fair amount of hydroxyl (phenolic and aliphatic) and carboxylic groups that own reactive hydrogen, lignin has the potential to replace polyols in polyurethane production. For example, polyurethane film was prepared from organosolv lignin with polyethylene glycol as co-polyol and soft segments [6] with or without catalyst [7]. Polyurethane foam was prepared from kraft lignin using polyethylene glycol as solvent [8]. Water-soluble lignosulfonate from sulfite pulping was used to prepare rigid polyurethane foams in glycols [9]. Lignin from straw steam explosion was also investigated for polyurethane preparation [10]. A polyurethane elastomer (film) was prepared from flax soda lignin with polyethylene adipate and ethylene glycol as co-polyol and soft segment, but the resultant polyurethane film was heterogeneous and did not have adequate mechanical strength for any application when lignin content was over 10% (wt.) [11]. Because of the solid state and less accessible hydroxyl groups of lignin, chemical modification such as oxypropylation with alkylene oxide was proposed to improve the accessibility of the hydroxyl groups, which could convert lignin into liquid polyol with extended chain and exposed hydroxyl groups [5,12]. As a follow-up, recently, liquid polyol from oxypropylated pine kraft lignin was used to prepare rigid polyurethane foam [13]. The same group also investigated the reinforcement of rigid polyurethane foam from oxypropylated ethanol organosolv lignin with cellulose nanowhiskers [14].

Organosolv ethanol process uses aqueous ethanol to extract lignin from lignocelluloses in the presence of small amount of inorganic acid as catalyst. It was developed in 1970s and commercialized in 1980s at pilot scale for producing pulp from hardwood for papermaking [15-17]. Recently, we reevaluated the organosolv process as a pretreatment method of woody biomass for cellulose ethanol production. It was found that the organosolv process was an effective pretreatment for both hardwood and softwood and the resultant cellulosic substrates had a ready digestibility with cellulases [18-21]. The isolated organosolv lignin during the pretreatment had attractive properties such as high purity, low molecular weight and narrow distribution, and more functional groups and the lignin was expected to have great potential in developing high-value lignin products [18,22]. However, the products and market of organosolv lignin have not been sufficiently developed. It is believed that the successful commercialization of organosolv pretreatment is greatly dependent on whether the organosolv lignin can be utilized efficiently and in value-added ways, which is expected to offset the high cost of the organosolv process.

In the present research, hardwood ethanol organosolv lignin (HEL) was evaluated to replace synthesized polyol to prepare rigid polyurethane foam and compared with hardwood kraft lignin (HKL). The effect of lignin addition on foam preparation (viscosity of polyols) and foam properties (density, compressive strength, and cellular structure) was investigated. Chain extenders (glycerol and butanediol) were examined for improving the properties of the lignin-based polyurethane foams.

## Results and discussion

### Effect of replacement of polyol by lignin on the preparation of rigid polyurethane foam

The content of functional groups and molecular weight of the HEL and HKL lignins are summarized in Table 1. HKL had more phenolic and aliphatic hydroxyl groups than HEL, suggesting that HKL should be more reactive as a polyol than HEL in polyurethane foam preparation. In addition, HKL had lower molecular weight than HEL. Therefore, it was expected that HKL might perform better in preparing polyurethane foams because of more functional groups (more crosslinking points) and low molecular weight (high mobility and low viscosity).

**Table 1:** Functional groups and molecular weight of the lignin samples

**Lignin**	**Ph-OH**	**Al-OH**	**MeO**	**M**_**w**_	**M**_**n**_	**M**_**w**_**/M**_**n**_
	**mmol/g**	**mmol/g**	**mmol/g**			
HEL	2.76	2.88	6.16	2600	1600	1.62
HKL	4.29	4.12	5.81	2400	1330	1.80

Note: HEL, hardwood ethanol organosolv lignin; HKL, hardwood kraft lignin; Ph-OH, phenolic hydroxyl group; Al-OH, aliphatic hydroxyl group; MeO, methoxyl group; M_w_, weight average molecular weight; M_n_, number average molecular weight.

**Figure 1:**
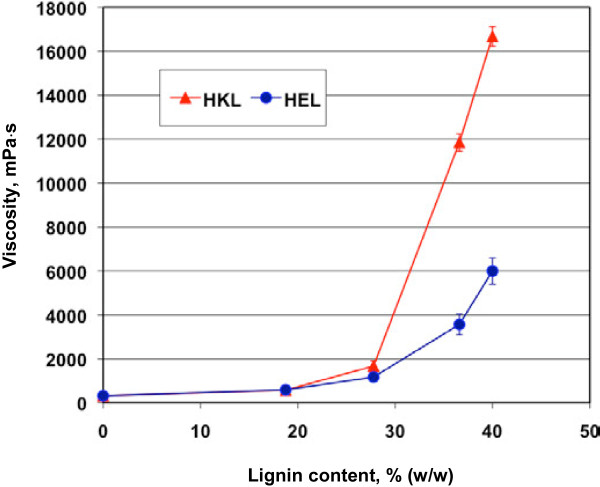
**Effect of lignin addition on the viscosity of polyether polyol (Voranol 270).** HKL, hardwood kraft lignin; HEL, hardwood ethanol organosolv lignin.

Viscosity of polyol is critical to the preparation of polyurethane foam and cellular structure of resultant foam. High viscosity could cause problems when mixing the foam ingredients and affect the generation and distribution of the bubbles/cells formed by the CO_2_ from the reaction between blowing agent (water in this study) and polydiisocyanate. The effect of blending the lignins in polyether polyol (Voranol 270) on viscosity is shown in Figure 1. In general, blending the lignins in Voranol 270 increased the viscosity of the polyol. When lignin addition was less than 28% (w/w in the polyol), the viscosity increased slowly. For example, 28% lignin elevated the viscosity from approximately 400 mPa·s of pure Voranol 270 to 1,600-1,800 mPa·s of the mixture of lignin and the polyol. However, the viscosity jumped sharply when lignin addition was more than 28% (w/w), in particular when HKL was added. For example, 40% lignin resulted in a viscosity of 6,000 or 16,700 mPa·s for HEL or HKL, respectively. As shown in Figure 1, HKL caused a much higher viscosity increase than HEL did, although the former had lower molecular weight than the latter (Table 1). This could be attributed to the better solubility/miscibility of HEL in polyol. HEL isolated from ethanol organosolv process was fairly soluble in ethanol and thereby had good miscibility and dispensability in the polyol (polyalcohol), while HKL produced from kraft pulping was insoluble in alcohols and was just suspended in the polyol, which resulted in a high viscosity.

**Figure 2:**
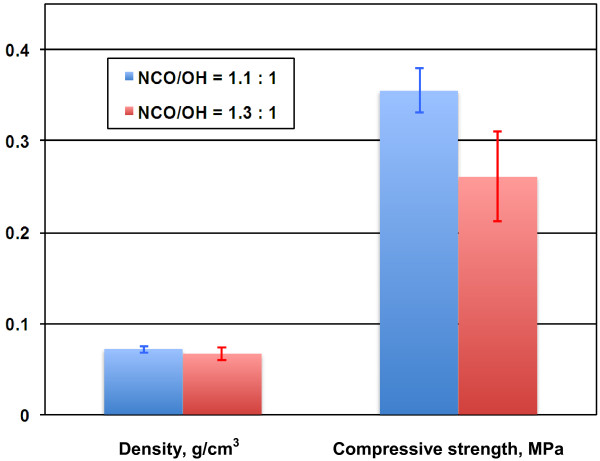
**Effect of NCO/OH ratio on the properties of lignin-based rigid polyurethane foam.** Foam formula: HEL lignin, 50% HEL and 50% Voranol 270 (molar percentage).

One of the most important parameters in polyurethane foam preparation is the molar ratio of isocyanate to hydroxyl groups (NCO/OH). A suggested NCO/OH ratio is 1.1:1 for rigid foam [1], and the excessive isocyanate is for reacting with blowing agent (water) to generate CO_2_ and form bubbles and cellular structure of the polyurethane foam. To investigate the effect of NCO/OH ratio on lignin-based polyurethane foam, lignin-containing foams were prepared at two NCO/OH ratios (1.1 and 1.3:1). As expected, the foams prepared at 1.3:1 NCO/OH ratio had more bubbles than the foams at 1.1:1 ratio because the excessive MDI reacted with water and formed more carbon dioxide, which resulted in more and larger bubbles. They did not significantly affect the density (only slightly decreased), as shown in Figure 2. However, since the larger and irregular bubbles resulted in less uniform cellular structure of the foam, the compressive strength decreased significantly when the ratio of NCO/OH increased from 1.1:1 to 1.3:1.

**Figure 3:**
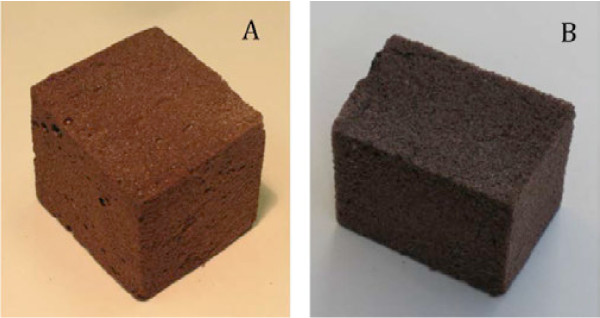
**Rigid polyurethane (PU) foams containing lignins.A**: PU foam containing 50% hardwood ethanol organosolv lignin (HEL); **B**: PU foam containing 50% hardwood kraft lignin (HKL).

**Figure 4:**
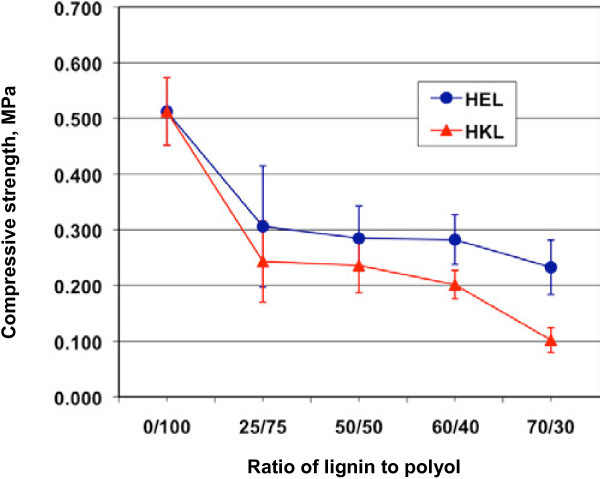
**Effect of lignin addition on the compressive strength of rigid polyurethane foams.** Ratio of lignin to polyol, molar ratio of the hydroxyl groups from lignin to those from polyol (Voranol 270); HEL, hardwood ethanol organosolv lignin; HKL, hardwood kraft lignin.

Representative pictures of polyurethane foams containing HEL or HKL lignin are shown in Figure 3. The foams appeared the brown color of lignin, and the HEL-containing foam had a lighter color than the HKL-containing one because HEL was lighter than HKL in color. Both foams had uniform cellular structure, but the HEL-containing foam felt tougher and stronger than the HKL-containing one, which was in agreement with the results of compressive strength in Figure 4.

To verify whether the lignin was chemically crosslinked or just physically trapped in the polyurethane foam, the foam prepared with 25% (w/w) HEL was extracted with 90% dioxane (dioxane/water, v/v), a good solvent of HEL lignin. In the experiment, the foam was cut into small pieces of approximately 5 × 5 mm and extracted with the dioxane in a Soxhlet extractor for 24 hours to see the weight loss of the foam. Pure polyurethane foam without lignin was used as reference. It was found that the pure polyurethane foam lost approximately 3% of its original weight during the extraction, while the HEL-containing foam lost 7%. The results indicated that although more material was extracted from the lignin-containing foam, the majority of the lignin was not extractable, suggesting that the lignin be chemically cross-linked not physically trapped in the foam.

### Effect of replacement of polyol by lignin on the density of polyurethane foam

**Figure 5:**
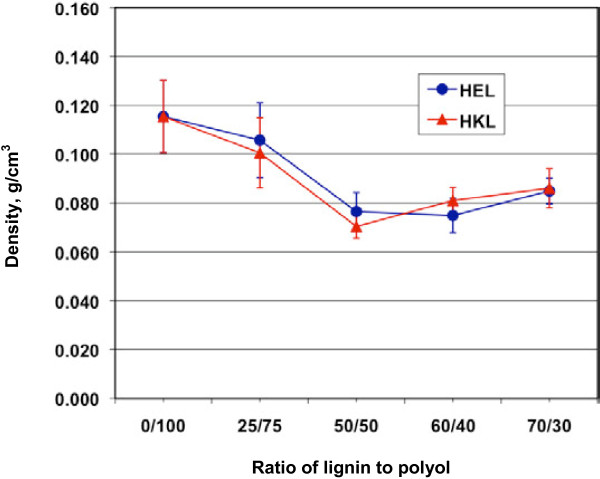
**Effect of lignin addition on the density of rigid polyurethane foams.** Ratio of lignin to polyol, molar ratio of the hydroxyl groups from lignin to those from polyol (Voranol 270); HEL, hardwood ethanol organosolv lignin; HKL, hardwood kraft lignin.

**Figure 6:**
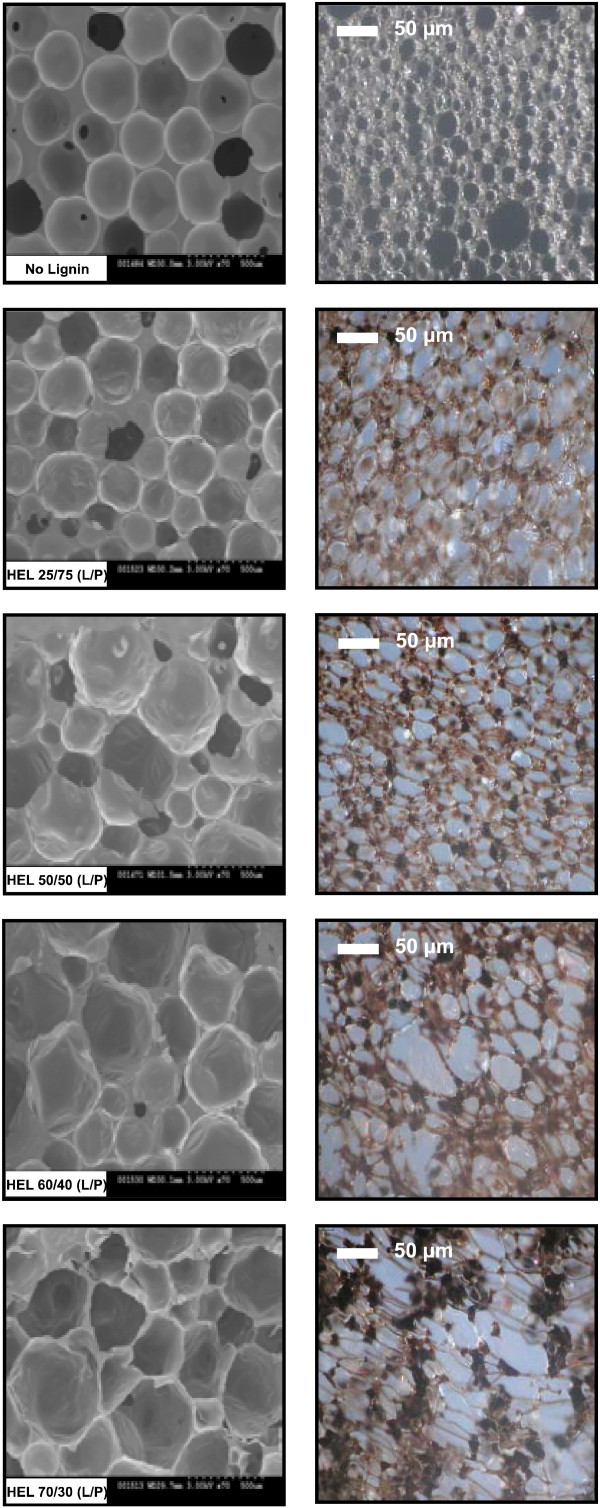
**Effect of lignin addition on cellular structure of rigid polyurethane foams.** HEL, hardwood ethanol organosolv lignin; L/P, lignin/polyol (Voranol 270).

As shown in Figure 5, the addition of lignin reduced the density of the foams, which is actually desirable if the foam is used as packing or insulation material. The density of pure polyurethane foam was about 0.116 g/cm^3^, and decreased by 30% when the polyol was replaced by 50% with lignin. This was probably because the lignin addition made the cellular structure of the foam less uniform and formed more larger cells (bubbles), as discussed above, which reduced the mass per unit volume of the foam and thereby the density. However, further increasing lignin content reversely resulted in a slightly higher density, likely because too much lignin affected the uniformity of the cells and part of the lignin was even not well dispersed in the foam and assembled together as big granules, which reduced the void volume and increased density. These were in agreement with the observations of cellular structure of the foams shown in Figure 6. It is apparent that the two types of lignin did not shown significant difference in terms of foam density.

### Effect of replacement of polyol by lignin on the compressive strength of polyurethane foam

Replacing the polyol with 25% lignin reduced the compressive strength of the foam by 40%, compared to pure polyurethane foam without lignin, as shown in Figure 4, primarily because (1) the lignin was less reactive (hydroxyl groups in lignin was less accessible) than the polyol Voranol 270, and therefore the crosslinking density and strength of lignin-containing foam was lower than those of the pure PU foam; (2) the lignin was not completely miscible with the polyol, and thereby the lignin was not uniformly dispersed in the foam; and (3) the introduction of lignin reduced the uniformity of the foam cellular structure, and the deficiency in the cellular structure weakened the stability and strength of the structure.

Further increasing lignin content from 25% to 60% did not result in additional drop of the strength, but when lignin content was more than 60%, the compressive strength decreased again because too much lignin resulted in more irregular cellular structure and weakened the crosslinks, as shown in Figure 6.

It was also seen from Figure 4 that the foams containing HEL had higher compressive strength than those containing HKL. Better miscibility of HEL with the polyol over HKL was likely the reason. As discussed above, poor miscibility of HKL with the polyol resulted in poor dispersion of the lignin in the foam and therefore fewer and weaker chemical crosslinking between the lignin and MDI. It should be pointed out that HKL had more hydroxyl groups than HEL (Table 1), and therefore at the same molar ratio of lignin to polyol, the foam with HEL actually had more lignin by weight than the foam with HKL. As compared in Table 2, the HEL foam had approximately 30% more lignin than HKL foam. Considering this fact, HEL foam actually had much higher compressive strength than HKL foam at the same lignin content.

**Table 2:** Lignin content in rigid polyurethane foams

**Ratio of lignin to polyol**	**25/75**	**50/50**	**60/40**	**70/30**
HKL in PU foam, % (w/w)	9	19	23	28
HEL in PU foam, % (w/w)	12	25	30	36

Note: Ration of lignin to polyol, molar ratio of the hydroxyl groups from lignin to those from polyol (Voranol 270). HKL, hardwood kraft lignin; HEL, hardwood ethanol organosolv lignin; PU, polyurethane.

### Cellular structure of lignin-based polyurethane foam

As shown in Figure 6, cellular structure of the HEL-containing rigid polyurethane foams was observed under scanning electron microscope (SEM, images in the left column) and light microscope (images in the right column). Pure polyurethane foam without lignin had uniform cell size and regular cell shape, and it looked semitransparent with a light yellow color. With the introduction of HEL, the foam turned to the brown color of lignin. In addition, the shape of the cells became less regular, and large cells formed as well. It seemed that the effect of lignin on the cellular structure of the foams was insignificant when lignin replacement was less than 50%. However, when lignin ratio increased to 60% in particular to 70%, the foam cells became significantly irregular and many large cells (bubbles) formed. Furthermore, with the increased lignin content, lignin became poorly dispersed in the foam, and many large lignin granules were clearly visible under light microscope. The irregular cells, large bubbles, and poorly dispersed lignin were likely responsible for the low compressive strength of the foams at high lignin content, as discussed above. The cellular structures of HKL foams (images are not provided) were similar to those of HEL foams, but more irregular.

### Effect of chain extenders on properties of lignin-containing polyurethane foam

**Figure 7:**
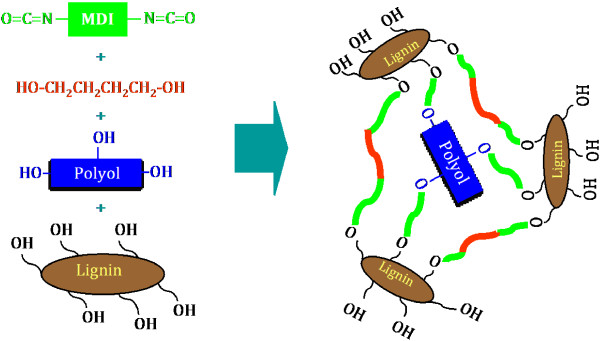
Illustration of the function of chain extender (butanediol) in polyurethane foam.

**Figure 8:**
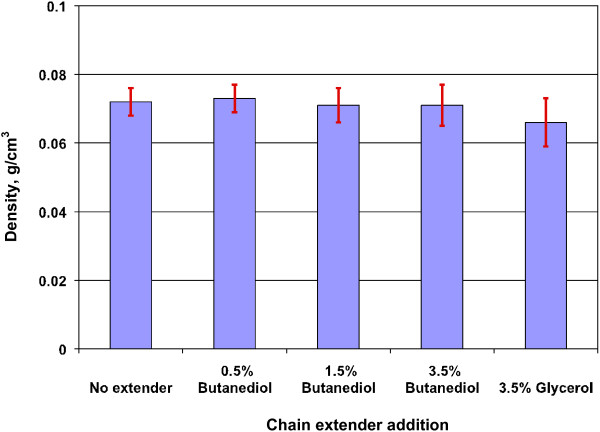
**Effect of extenders on the density of lignin-containing rigid polyurethane foams.** Foam formula: HEL lignin, 50% HEL and 50% Voranol 270 (molar percentage).

The results above clearly indicated that replacing polyol with the lignins negatively affected the strength and structure of rigid polyurethane foams. This was partially due to the low hydroxyl groups content of the lignins and the poor accessibility of the groups. Chain extender is supposedly able to solve the problem and improve the performance and properties of lignin-containing foams. Chain extenders generally have low molecular weight and are bifunctional compounds for enhancing the crosslinking in polyurethane foams. Glycerol and 1,4-butanediol are common chain extenders in polyurethane foam formulation. The function of chain extenders in the preparation of lignin-containing polyurethane foam is illustrated in Figure 7. The effect of the chain extenders on density is shown in Figure 8. It can be seen that the density values did not significantly changed when more chain extender (butanediol) was added. This suggested that chain extender did not substantially affect foam structure (cell amount, size and distribution) when the ratio of NCO/OH was kept constant.

**Figure 9:**
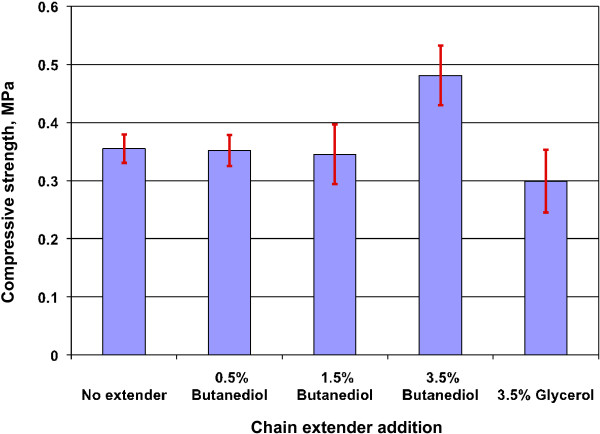
**Effect of extenders on the compressive strenght of lignin-containing rigid polyurethane foams.** Foam formula: HEL lignin, 50% HEL and 50% Voranol 270 (molar percentage).

However, the addition of chain extender, such as 3.5% butanediol, improved the compressive strength of the foam, as shown in Figure 9, because the chain extender increased the accessibility of hydroxyl groups in lignin. At lower loading percentages, butanediol did not have substantial effect on compressive strength improvement, probably because the extender molecules were not enough to improve the crosslink between MDI and lignin. Glycerol was not as effective as butanediol as chain extender, presumably because the three hydroxyl groups of glycerol consumed more MDI than butanediol, thereby reducing the crosslinking density between MDI and lignin and consequently the strength of the foam.

## Conclusion

Polyol was replaced with hardwood ethanol organosolv lignin (HEL) or hardwood kraft lignin (HKL) from 25% to 70% (molar percentage) in preparing rigid polyurethane foam (RPF). The prepared foams contained 12-36% (w/w) HEL or 9-28% (w/w) HKL. The density, compressive strength, and cellular structure of the foams were investigated and compared. It was found that the majority of the lignin was chemically crosslinked not just physically trapped in the foams as filler. The foams had satisfactory structure and strength up to 25-30% (w/w) HEL or 19-23% (w/w) HKL addition. The results indicated that HEL performed much better in RPFs and was able to give a better strength at the same lignin content or replace more polyol at the same strength than HKL presumably because the former had a better miscibility with the polyol than the latter. Addition of chain extender such as butanediol could improve the strength of lignin-containing RPFs.

## Methods

### Materials

Hardwood organosolv ethanol lignin (HEL) was generously provided by Lignol Innovation (Vancouver, Canada), produced from mixed hardwoods using the organosolv ethanol process [23]. Hardwood kraft lignin (HKL) was generously contributed by Westvaco (Covington, VA), which was prepared from the black liquor of mixed hardwoods kraft pulping [24]. Both lignins were spray-dried and had uniform and fine particle size, and HEL was slightly light in color (both brown) than HKL. The lignins were dried in a 105°C oven overnight before used in preparing polyurethane foam.

**Scheme 1:**
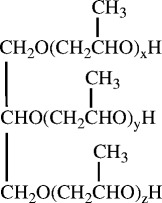
Structure of polyether polyol (Voranol 270).

Polymeric MDI (Methyl Diphenyl Diisocyanate, PAPI 27, isocyanate content 7.5 mmol/g) and polyether polyol (Voranol 270, polyether triol, molecular weight 700, hydroxyl content 4.3 mmol/g) were generously provided by DOW Chemicals (Toronto, Canada). The structure of Voranol 270 is shown in Scheme 1. Polyether-modified polysiloxane (Tegostab BF 2370) as surfactant and Tin-(II)-isooctoate (Kosmos 29) as catalyst were generously provided by Goldschmidt Chemical (McDonald, PA). All these commercial products were used as received without any modification or pretreatment. Other chemicals were purchased from Sigma-Aldrich (St. Louis, MO) and used as received.

### Characterization of the lignins

The functional groups of HEL and HKL were estimated using ^1^H NMR, and molecular weight was estimated using gel permeation chromatography (GPC). In brief, Functional groups (phenolic hydroxyl, aliphatic hydroxyl, and methoxyl groups) were determined using ^1^H-NMR. Lignin acetate (50 mg) and 5 mg of *p*-nitrobenzaldehyde (NBA, internal standard) were dissolved in 0.5 mL of deuterochloroform, and ^1^H-NMR spectra were recorded on a Bruker AV-300 spectrometer. The functional groups were estimated from the areas of their peaks, referring to the proton peak area of NBA [25]. The number average and weight average molecular weights (M_n_ and M_w_, respectively) of HEL and HKL were estimated by GPC using a Waters (Rochester, MN) HPLC system equipped with a Waters 717 autosampler, a Waters 2410 refractive index detector, and three Waters Styragel columns (HR5E, HR4, and HR2) in tandem. Lignin acetate (0.5 mg) was dissolved in 1 mL of tetrahydrofuran, and 30 μL of the solution were injected. The columns were calibrated with polystyrene standards [18].

### Preparation of polyurethane foam from lignin

Lignin, polyol (Voranol 270), blowing agent (water), surfactant (Tegostab BF 2370), and catalyst (Kosmos 29) were weighed into a container (polystyrene foam cup) according to preset foam formula. The ingredients were first thoroughly mixed manually using a glass rod to disperse lignin in the polyol. When the pre-determined MDI was added into the container, the mixture was stirred at high speed using a kitchen egg beater for 20 seconds, and left in a fume hood at room temperature to allow the foam rising. The prepared foam was kept at room temperature in the hood for one week for curing and aging before characterization. Polyurethane foam without lignin was prepared as reference following the same procedure above. All the foams were prepared in five duplicates, and the average of the results from the five samples was reported. The amount of lignin, polyol and MDI were determined according to the desired lignin content to add and the molar ratio of isocyanate to hydroxyl (NCO/OH). The NCO/OH ratio was calculated using the equation below:

$$\frac{\mathrm{NCO}}{\mathrm{OH}}=\frac{W_{\mathrm{MDI}}{\left[\mathrm{NCO}\right]}_{\mathrm{MDI}}}{W_{L}{\left[\mathrm{OH}\right]}_{L}+W_{P}{\left[\mathrm{OH}\right]}_{P}}$$

Where, W_MDI_, W_L_ and W_P_ = weights (g) of MDI, lignin and polyol, respectively; [NCO]_MDI_ = molar content of isocyanate groups in MDI; [OH]_L_ and [OH]_P_ = molar content of total hydroxyl groups in the lignin and the polyol, respectively.

#### Viscosity

Viscosity of the mixture of the polyether polyol (Voranol 270) and lignin (HEL and HKL) was determined using a Brookfield dial reading rotary viscometer (Model LVT). The reported viscosity was the average of five measurements.

#### Characterization of polyurethane foams from lignin

Density of the foams was measured from the weight and volume of foam samples. Compressive strength was determined on an MTS Sintech 30/D material testing machine according to ASTM D-1621 (Standard test method for compressive properties of rigid cellular plastics). Light microscope images of the foams were taken on an Olympus BX51 microscope. SEM images of the foams were taken on a Hitachi S-2600N variable pressure scanning electron microscope.

## Abbreviations

GPC: Gel permeation chromatography; HEL: Hardwood ethanol organosolv lignin; HKL: Hardwood kraft lignin; HPLC: High-Performance Liquid Chromatography; MDI: Methylene diphenyl diisocyanate; M_n_: Number average molecular weight; M_w_: Weight average molecular weights; NBA: *p*-nitrobenzaldehyde; NMR: Nuclear magnetic resonance; RPF: Rigid polyurethane foam; SEM: Scanning electron microscope; TDI: Toluene diisocyanate.

## Competing interests

The authors declare that they have no competing interests.

## Authors’ contributions

XP and JNS designed research; XP performed research; XP and JNS analyzed data and wrote the paper. All authors read and approved the final manuscript.

## Authors’ information

XP is an Associate Professor of Bioenergy and Biomaterials. XP’s areas of interest include pretreatment and fractionation of lignocellulose, chemical and enzymatic saccharification of lignocellulose, biofuels (e.g. ethanol and hydrocarbon) from lignocellulose, and cellulose, hemicellulose and lignin based materials. JNS is a Professor of Forest Products Biotechnology. JNS’ research interests are application of enzymes in enhancing pulp and fiber properties, fiber modification and bleach boosting pulps, bioconversion of lignocellulosic residues to ethanol, microbiology of wastewater treatment, application of fungi to upgrading and modification of forest products, pulp and paper and waste streams.
